# Evaluation of factors associated with complications in amoebic liver abscess in a predominantly toddy‐drinking population: A retrospective study of 198 cases

**DOI:** 10.1002/jgh3.12183

**Published:** 2019-05-30

**Authors:** Ashish K Jha, Praveen Jha, Madhur Chaudhary, Shubham Purkayastha, Sanjeev K Jha, Ravish Ranjan, Rajeev N Priyadarshi, Ramesh Kumar

**Affiliations:** ^1^ Department of Gastroenterology Indira Gandhi Institute of Medical Sciences Patna India; ^2^ Department of Radiology All India Institute of Medical Sciences Patna India; ^3^ Department of Gastroenterology All India Institute of Medical Sciences Patna India

**Keywords:** amoebic liver abscess, complication, *Entamoeba histolytica*, interventional therapy, palm wine, prognosis, tari, toddy

## Abstract

**Background and Aim:**

Although the mortality rate has declined in recent years, amoebic liver abscesses (ALAs) still carry a substantial risk of morbidity. Studies regarding the indicators of severity, complication, or prognosis of ALA are limited in number and heterogeneous in methodology and results.

**Methods:**

Clinicodemographic profile, therapeutic modalities, and outcomes of indoor ALA patients admitted between January 2016 and October 2017 were analyzed. An analysis of possible prognostic factors associated with complications and interventional therapy in patients with ALA was performed retrospectively.

**Results:**

Data of 198 patients with ALA (mean age: 45 ± 12.1; M:F ratio: 193:5) were analyzed. The volume of abscess (503.1 ± 391.2: 300.2 ± 305.8 mL), elevated liver enzymes, and duration of hospital stay (11.98 ± 5.75): 10.23 ± 4.1 days) were significantly (*P* < 0.05) higher in alcoholic, compared to nonalcoholic, individuals. On univariate analysis, older age, duration of alcohol consumption, smoking, leukocytosis, hyperbilirubinemia, hypoalbuminemia, hyponatremia, and a larger volume of abscess were found to be significantly (*P <* 0.05) associated with complications. On multivariate analysis, older age, duration of alcohol consumption, smoking, leukocytosis, hyperbilirubinemia, hypoalbuminemia, and hyponatremia were found to be significantly (*P <* 0.05) associated with complications. Male gender, hypoalbuminemia, and larger volume of abscess were significantly (*P* < 0.05) associated with interventional treatment.

**Conclusion:**

Older age, leukocytosis, hyperbilirubinemia, hypoalbuminemia, hyponatremia, chronic alcoholism, and smoking are independent factors significantly associated with complications in patients with ALA. Hypoalbuminemia, larger volume of abscess, and male gender are independent variables associated with the requirement of interventional therapy.

## Graphical abstract

Studies regarding the evaluation of factors associated with complications in amoebic liver abscess (ALA) in a predominantly toddy‐drinking population are limited in number. Older age, leukocytosis, hyperbilirubinemia, hypoalbuminemia, hyponatremia, chronic alcoholism, and smoking are independent factors significantly associated with complications in patients of ALA. Hypoalbuminemia, larger volume of abscess, and male gender are independent variables associated with the requirement of interventional therapy.

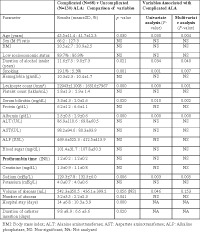

## Introduction

Amoebic liver abscess (ALA) is the most common extraintestinal form of invasive amoebiasis. As per a World Health Ogranization report, *Entamoeba histolytica* (EH) infections are prevalent throughout the tropical countries, with up to 50 million infections, and approximately 100 000 deaths occur each year, mostly from ALA.^1^ The incidence of ALA has been reported to vary between 3 and 9% of all cases of amoebiasis.^2^ ALA is still very common in tropical countries, especially in South Asian populations consuming toddy/tari (palm wine) or other indigenous alcoholic beverages.

The clinicodemographic profile, rate of complications, and mortality among patients with ALA vary in different parts of the world. ALA is associated with significant morbidity and mortality rates. Mortality among patients with ALA varies from 2% (recent data) to 18% (data from the 90s).^3^, ^4^, ^5^ About 20–40% of ALA patients are associated with complications.^4^, ^5^, ^6^ Although the mortality rate has declined in recent years, ALA still carries a substantial risk of morbidity. Assessment of the severity of disease and identification of factors associated with complications and prognosis are important for determining the appropriate level of care and choosing early intervention therapy. Studies regarding the indicators of severity, complication, or prognosis are very limited in number and heterogeneous in methodology and results.^7^, ^8^, ^9^, ^10^

Here, we studied the indicators of the risk of complications in patients with ALA. We further determined the variables associated with interventional therapy in patients with ALA.

## Methods

The study was conducted at a teaching hospital in eastern India. We retrospectively analyzed patients (>18 years of age) with ALA who were admitted in the gastroenterology unit between January 2016 and October 2017. An analysis of possible prognostic factors associated with complications and interventional therapy in patients with ALA was performed. The study was approved by the institute's ethical review committee. The aim of this study was to identify the indicators of risk of complications in patients with ALA. We also determined the variables associated with interventional therapy in patients with ALA.

A detailed history was taken regarding the clinical features, risk factors, and complications of ALA. A history of alcoholism in terms of type, duration, and quantity of daily alcohol intake was obtained. A history of toddy/tari (palm wine) ingestion, which is very common in this part of India, was obtained. “Alcoholism” was defined as per the CAGE (cut‐annoyed‐guilty‐eye) questionnaire.^11^ Alcohol consumption in excess of 60 g per day was considered significant in our study. Dietetic and nutrition assessments were performed. Patients were considered to be undernourished when either their body mass index (BMI) was <18.5 kg/m^2^ or their serum albumin was <3.5 g/dL. Patients were divided into three socioeconomic classes (modified Kuppuswamy's scale): upper, middle, and lower.^12^

All patients underwent routine blood tests and tests for serum antibodies against the *EH* antigen. Stool examination was performed for cysts of *EH*. Ultrasonography (USG) and chest x‐ray were performed in all patients if not already done. Abdominal and thoracic computed tomography (CT) scan and magnetic resonance cholangiopancreatography and colonoscopy were performed if indicated clinically. USG‐guided fine needle aspiration (FNA) was performed to obtain pus for microscopy, culture and sensitivity, and drainage of abscess. Diagnosis was based on the characteristic clinical (presence of fever, pain, or tenderness in the right upper abdomen) and radiological findings (focal round or oval hypoechoic liver lesions in USG/CT) in the presence of anchovy sauce appearance of pus or the presence of serum antibodies against *EH* antigen or visualization of *EH* trophozoites in pus. Complications considered were intra‐abdominal rupture, pleural rupture and hydrothorax/pyothorax, multiple organ dysfunction, sepsis, biliary communications with abscess cavity, bronchobiliary fistula, cholangitis, pericardial rupture, vascular complications, and death. Asymptomatic sympathetic effusion or ascites (prerupture syndrome) was not considered a complication.

Medical therapy mainly consisted of analgesics, amoebicidal agents, and supportive therapy. All patients received oral or intravenous metronidazole, 40 mg/kg daily, in three divided doses for 10–14 days. Bacterial coinfections were treated with antibiotics. Radiological interventions (percutaneous needle aspiration [PNA] or percutaneous catheter drainage [PCD]) were performed in the presence of abscess size >5 cm, left lobe abscess, no response to medical therapy at the end of 48 h, and/or impending rupture (<1 cm liver tissue between abscess and liver margin).^13^, ^14^ Patients with an abscess size of less than 10 cm underwent PNA, whereas patients with abscess size of >10 cm were treated with PCD.^13^, ^14^

Surgery was considered in cases of ruptured liver abscess with multiseptate abdominal collection or failed radiological therapy. Pyothorax or hydrothorax was managed with chest tube drainage. Biliary stenting was performed in patients with complications of biliary obstruction, major biliary communications, or bronchobiliary fistula. Patients were followed as inpatients during hospitalization and were re‐examined 1 month after discharge.

### *Statistical analysis*

All results are expressed as mean ± SD, median (range), or frequency (%) as appropriate. Quantitative variables, expressed as means ± SD, were compared using the Student *t*‐test. The association between two categorical variables was tested using the Chi‐square test or Fisher's exact test, wherever appropriate. The data for the groups were compared using a one‐way anova for continuous data. A *P*‐value of <0.05 was considered statistically significant. Data were analyzed using IBM SPSS Statistics for Windows software, version 22.0 (IBM Corp., Armonk, NY, USA).

## Results

**Figure 1:**
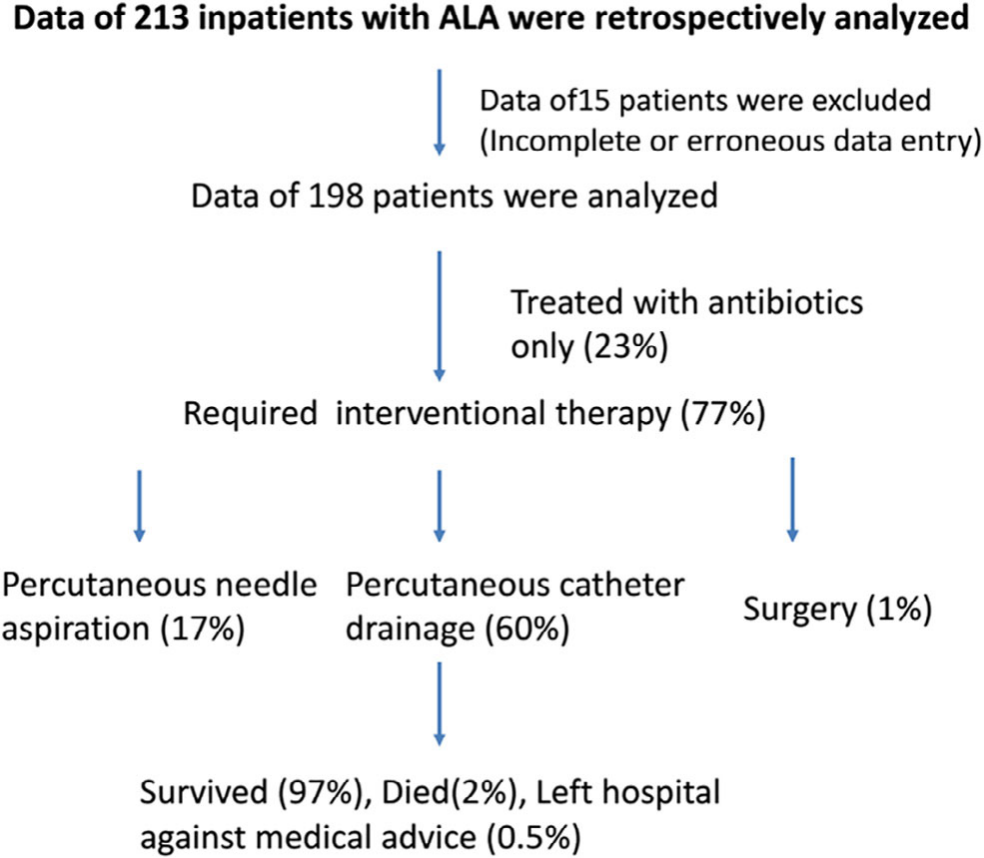
Flow chart showing the screening of ALA patients and their management and outcomes.

During the study period, we screened the data of 213 patients with ALA and included 198 patients in our study (mean age: 45 ± 12.1; M:F ratio: 193:5). Clinical and investigational profiles of ALA patients and their management and outcomes are summarized in Fig. 1, Tables 1 and 2.

**Table 1:** Demographic and clinical features of amoebic liver abscess patients (*n* = 198)

Age (mean ± SD)	45 ± 12.1 years; (M:F ratio:193:5)
Risk factors	Low socioeconomic status: 174 (88%); alcoholism: 168 (85%); hypoalbuminemia: 168 (85%)
Symptoms	Pain: 193 (97%); fever: 184 (93%); dyspnea: 19 (10%); diarrhea 10 (5%); per rectal bleeding 6 (3%); altered sensorium 4 (2%)
Sign	Hepatomegaly 157 (79%); jaundice 55 (28%); pedal edema 34 (17%); abdominal distension 31 (16%)
Complications	68 (34%)
Rupture	60 (30%) [peritoneum 47 (24%); pleural 13 (7%)]
Other complications	Ascites 55 (28%); multiple organ dysfunction 16 (8%); hydrothorax/pyothorax 21 (11%); biliary communication 6 (3%), bronchobiliary fistula 2 (1%), IVC thrombosis 1 (0.5%), death 4 (2%)

IVC, inferior vena cava.

**Table 2:** Baseline laboratory parameters (*n* = 198)

Hemoglobin (gm/dL)	10.4 ± 1.99
Leukocyte count (/mm^3^)	18 723 ± 9598
Platelet count (lakhs/mL)	2.92 ± 1.41
Serum bilirubin (mg/dL)	2.48 ± 3.41
Protein (g/dL)	6.4 ± 1.1
Albumin (g/dL)	2.77 ± 0.64
ALT (U/L)	75 ± 81
AST (U/L)	86 ± 94
ALP (IU/L)	432 ± 455
Prothrombin time	1.24 ± 0.24
Creatinine (mg/dL)	1.14 ± 0.75
Sodium (mEq/L)	131 ± 6.9
Potassium (mEq/L)	4 ± 0.67
Amoebic serology	Positive: 182 (92)
Pus culture	Positive 18 (11)
Ultrasonography	
Solitary: multiple	106 (54): 92 (46)
Lobes (right: left: both lobes)	152 (77): 12 (6): 34 (17)
Size (cm) of abscesses	8.79 ± 3.36
Average volume	472.2 mL (largest abscess: 2035 mL)

Results are expressed in (mean ± SD) and percentage.

ALP, alkaline phosphatase; ALT, alanine aminotransferase; AST, aspartate aminotransferase; INR, international normalized ratio.

### *Clinical and investigational profile*

The highest number of admissions was observed in May to August (48%), followed by January to April (29%) and September to December (23%). A history of alcoholism was present in 168 (85%) patients. Among alcoholics, 139 (70%) were toddy drinkers, and 29 (15%) were distilled alcohol drinkers; 174 (88%) patients belonged to low socioeconomic status. Most of the patients had evidence of malnourishment. Anemia and hypoalbuminemia were seen in 181 (91%) and 168 (85%) of patients, respectively. However, only 23 (12%) patients had low BMI. A history of chronic smoking was noted in 20 (10%) patients. Diabetes mellitus (DM) was present in five patients.

Pain was the most common symptom, being present in 193 (97%) patients, followed by fever 184 (93%). At least 68 (34%) of the ALA patients were admitted with complications. Rupture of liver in to the peritoneum and pleural cavity occurred in 60 (30%) patients (Table 1).

More than half of the patients had a solitary abscess (54%). Liver abscess was most commonly located in the right lobe (77%) (Table 2). Mean volume of the abscess was 472.2 mL. Trophozoites of EH in aspirated pus were observed in two patients. Pus culture showed growth of bacterial isolates in 18 (11%) patients. *Escherichia coli* (7), *Klebsiella* (3), *Acinetobacter* (3), *Pseudomonas* (2*), Staphylococcus* (2), and *Stenotrophomonas* (1) were isolated from pus. None of the pus samples cultured in Robinson's media were positive for *EH*. Amoebic serology was positive in 182 (92%) patients.

### *Treatment and outcome*

About one‐fourth of patients were managed with antibiotics. PNA and PCD were performed in 33 (17%) and 118 (60%) patients, respectively. Mean duration of PCD catheter insertion was 8.24 ± 6.99 days. Surgery was required in 1% of patients. All except five patients were discharged in satisfactory condition. Four (2%) patients with ruptured ALA died due to multiple organ dysfunction. A patient with a complication of acute respiratory distress syndrome was discharged against medical advice.

### *Comparison of patients with and without chronic alcoholism*

Age, BMI, hematological parameters, biochemical abnormalities, volume of abscess, and duration of hospital stay were compared between the alcoholic and nonalcoholic patients. Overall, the volume of abscess (503.1 ± 391.2: 300.2 ± 305.8 mL), elevated liver enzymes (alanine aminotransferase [U/L]—90.9 ± 9: 61.9 ± 53.1; aspartate aminotransferase [U/L]—90.9 ± 99.4: 61.9 ± 53.1; alkaline phosphatase [IU/L]—454.5 ± 482.4: 299.2 ± 186.8), and duration of hospital stay (11.98 ± 5.75: 10.23 ± 4.1 days) were significantly (*P* < 0.05) higher in alcoholic, compared to nonalcoholic, individuals. Among patients with complications, the serum alkaline phosphatase level (498.8 ± 556.4): 278.4 ± 137.4 [IU/L]) and volume of abscess (582.3 ± 349.4: 436.1 ± 399.2 mL) were significantly higher in alcoholic, compared to nonalcoholic, individuals (*P* < 0.05).

### *Comparison of patients with and without complications*

Clinical course and investigational profile were compared between ALA patients with or without complications. Individuals with complications had a longer duration of alcohol intake; older age; higher prevalence of smoking; higher leukocyte count; higher incidence of hyperbilirubinemia, hypoalbuminemia, and hyponatremia; greater number of abscesses; longer duration of PCD catheter insertion; and longer hospital stay than individuals who did not have complications (*P <* 0.05). The volume of abscess was larger in patients with complications compared to patients without complications (*P* = 0.056) (Table 3).

**Table 3:** Factors associated with complicated amoebic liver abscess(ALA)

	Complicated (*n* = 68) versus uncomplicated (*n* = 130) ALA: comparison of variables		Variables associated with complicated ALA	
Parameter	Results (mean ± SD, %)	*P*‐value	Univariate analysis (*P*‐value)	Multivariate analysis (*P*‐value)
Age (years)	45.5 ± 11.4: 41.7 ± 12.3	0.030	0.030	0.004
Gender (M:F) ratio	66:2: 127:3	NS	NS	NS
BMI	20.5 ± 2.7: 20.9 ± 2.5	NS	NS	NS
Low socioeconomic status	89.7%: 86.9%	NS	NS	NS
Duration of alcohol intake (years)	11.6 ± 7.5: 9.0 ± 7.3	0.021	0.034	0.040
Smoking	19.1%: 5.3%	0.001	0.001	0.007
Hemoglobin (gm/dL)	10.3 ± 2.3: 10.4 ± 1.7	NS	NS	NS
Leukocyte count (/mm^3^)	22 943 ± 11 005: 16 516 ± 7967	0.000	0.000	0.001
Platelet count (lakhs/mL)	2.8 ± 1.3: 2.9 ± 1.4	NS	NS	NS
Serum bilirubin (mg/dL)	3.3 ± 4.3: 2.0 ± 2.6	0.020	0.010	0.002
Protein (g/dL)	6.2 ± 1.2: 6.6 ± 1.1	NS	NS	NS
Albumin (g/dL)	2.5 ± 0.5: 2.9 ± 0.6	0.000	0.000	0.000
ALT (U/L)	86.9 ± 110.6: 69.8 ± 60.5	NS	NS	NS
AST(U/L)	98.2 ± 94.6: 80.3 ± 93.9	NS	NS	NS
ALP (IU/L)	469.6 ± 525.3: 412.5 ± 413.9	NS	NS	NS
Blood sugar (mg/dL)	101.4 ± 31.7: 107.8 ± 50.3	NS	NS	NS
Prothrombin time	1.2 ± 0.2: 1.2 ± 0.2	NS	NS	NS
Creatinine (mg/dL)	1.3 ± 0.9: 1.1 ± 0.8	NS	NS	NS
Sodium (mEq/L)	129.3 ± 7.8: 132.5 ± 6.0	0.006	0.003	0.005
Potassium (mEq/L)	4.0 ± 0.7: 4.0 ± 0.6	NS	NS	NS
Volume of abscess (mL)	542.3 ± 350.5: 436.1 ± 399.2	0.056 (NS)	0.046	0.153
Number of abscess	3.2 ± 3.5: 2.2 ± 2.3	0.041	NS	NS
Hospital stay (days)	14 ± 6.8: 10.3 ± 3.9	0.000	NA	NA
Duration of catheter insertion (days)	9.8 ± 8.9: 6.6 ± 3.6	0.020	NA	NA

ALP, alkaline phosphatase; ALT, alanine aminotransferase; AST, aspartate aminotransferase; BMI, body mass index; ; INR, international normalized ratio; NA, not analyzed; NS, non‐significant.

### *Variables associated with complications*

In the univariate analysis, older age, longer duration of alcohol consumption, smoking, leukocytosis, hyperbilirubinemia, hypoalbuminemia, hyponatremia, and larger volume of abscess were found to be significantly (*P <* 0.05) associated with complications. In the multivariate analysis, older age, longer duration of alcohol consumption, smoking, leukocytosis, hyperbilirubinemia, hypoalbuminemia, and hyponatremia were found to be significantly (*P <* 0.05) associated with complications (Table 3). No association can be seen between the type of alcohol (toddy/tari or distilled alcohol) and complications.

### *Variables associated with requirement of interventional therapy*

Comparisons were also made between patients treated with antibiotics only and those who required interventional therapy. Multivariate analysis showed male gender, hypoalbuminemia, and larger volume of abscess to be significantly associated with interventional treatment (radiological and surgery) (*P* < 0.05).

## Discussion

There are wide variations in the clinicodemographic profile of ALA from different parts of the world.^13^, ^14^, ^15^, ^16^, ^17^, ^18^ Studies defining the indicators of severity are scarce from this part of world, where most of the ALA patients are associated with a history of toddy consumption. In the current study, we assessed the clinicodemographic profile of ALA patients in a predominant toddy‐drinking population of eastern India. We also identified the variables associated with complications of ALA.

Male gender, age (third to fifth decades), alcohol, low socioeconomic status, poor living and sanitary conditions, overcrowding, poor sanitation, unhygienic practices, malnourishment, immunosuppressed status, and DM are considered predisposing factors for ALA. The mean age in this study cohort was (45 ± 12.1) years; almost all the patients were males. These findings are almost similar to those of other studies from endemic regions, where patients with ALA usually presented in their third to fifth decade of life with a predominant male preponderance.^13^, ^14^, ^15^, ^16^

More than two‐thirds of patients in this study cohort were alcoholics. The volume of abscess, liver enzymes level, and duration of hospital stay were significantly (*P <* 0.05) higher in alcoholic, compared to nonalcoholic, patients. Alcohol consumption was also associated with a higher rate of complications in patients with ALA. Therefore, our study supports earlier studies that alcohol increases the prevalence and severity of ALA. Apart from age, gender, alcohol consumption, and malnutrition (hypoalbuminemia), low socioeconomic status was a major risk factor for ALA in our study cohort. Unlike alcoholism and malnutrition (hypoalbuminemia), low socioeconomic status was not independently associated with complications of ALA. The following mechanisms have been proposed for the predisposition of ALA by alcohol: (i) alcohol suppresses the function of Kupffer cells, which has the important role of clearing the amoeba^14^; (ii) hepatocyte damage by alcohol; (iii) hepatic accumulation of iron^19^; (iv) the invasive capacity of EH is facilitated by alcohol^20^; (v) nutritional deficiencies in alcoholics leading to lowered body resistance and suppression of liver function^20^; (vi) alcohol depress activity of immunity^16^; and (vii) entry of *EH* into the blood facilitated by dysbiosis of intestinal bacteria and alcohol‐induced intestinal hyperpermeability.^21^

Clinical features and treatment outcomes in our study cohort are nearly consistent with other studies from this region.^13^, ^14^, ^15^, ^16^ More than one‐third of patients had complications, mainly rupture of abscess cavity in to the peritoneum and pleural cavity. Pus culture showed bacterial superinfection in 11% of patients. The data of these patients were carefully analyzed to rule out pyogenic infection. Secondary bacterial infection is a common complication of ALA (10–20%).^22^ About two‐thirds of patients in this study cohort were treated with interventional therapy. A criterion for aspiration was the presence of abscess size >5 cm.^13^, ^14^ This explains the high rate of intervention in this study.

Studies regarding the indicators of severity or prognosis are heterogeneous in methodology and results. In a retrospective study (*n* = 125) by Chuah et al., diabetes mellitus, jaundice, hemoglobin and serum bilirubin levels, and pulmonary involvement were significantly associated with severe liver abscess.^7^ In a prospective study from India (*n* = 135), a bilirubin level > 3.5 mg/dL, encephalopathy, volume of abscess cavity, hypoalbuminemia (serum albumin level < 2.0 g/dL), and number of abscesses were identified as independent risk factors of mortality.^3^ However, this study was conducted in the late 90s, and the overall mortality in this study was high (18%). In another study from South India (*n* = 100), complications were noted in 24% of patients with a liver abscess. History of alcoholism (>10 years), prothrombin time (INR) > 1.7, leukocytosis (total leukocyte count >20 000/cc), and pleural effusion were demonstrated to be predictors of complication occurrence.^8^ However, this study included all cases of liver abscess (both pyogenic and amoebic). In a retrospective study (*n* = 140), Muñoz et al. found that hemoglobin, hematocrit, prothrombin time, total proteins, albumin, lactate dehydrogenase, and blood urea nitrogen were more altered in ALA patients who presented with complications.^9^

In the current study, older age, leukocytosis, hyperbilirubinemia, hypoalbuminemia, hyponatremia, longer duration of alcohol consumption, and smoking were independent factors significantly associated with complicated ALA compared to uncomplicated patients. The volume of abscess was larger in patients with complications compared to patients without complications (*P* = 0.056). The volume of abscess was significantly associated with complications in univariate analysis but did not reach statistical significance on multivariate analysis. All the aforementioned parameters, except hyponatremia and smoking, were described previously as indicators of complications in ALA.^8^ In the present study, other factors like hyponatremia and smoking were also found to be associated with complications. Besides baseline hyponatremia, hyponatremia in inpatients with ALA can be due to diuretics use for anasarca or through hypotonic intravenous fluid therapy. The association between hyponatremia and poor patient outcome has been demonstrated in numerous studies. A meta‐analysis showed that hyponatremia is associated with poor outcomes in patients affected by multiple disease types across large numbers of hospitalized patients (relative risk (RR) = 2.48 [2.09–2.95]).^23^ Whether hyponatremia is merely a marker or also a mediator of adverse patient outcomes remains to be elucidated. Our study cohort consisted of hospitalized patients only; this can explain the observed association between hyponatremia and complicated ALA.

Hyperbilirubinemia was noted in 27.8% of our study cohort. It was identified as an independent factor associated with complicated ALA. Hyperbilirubinemia is commonly seen in ALA due to compression or destruction of intrahepatic biliary channels. The result of our study is consistent with the study conducted by Datta et al. Jaundice was seen in 28% of patients, and raised bilirubin was associated with a higher rate of complications and mortality in patients with ALA than those without jaundice.^10^

Size of abscess was a criterion for intervention in this study. Hypoalbuminemia and larger volume of abscess were other factors independently associated with the requirement of interventional therapy in the current study. Male gender was also associated with interventions, but all except six patients were male; therefore, the possibility of a statistical error cannot be ruled out. In a recent study, large abscess volume (>500 mL or >10–8 cm), low albumin level, and high alkaline phosphatase level were significantly (*P* < 0.05) associated with failure of medical treatment of ALA.^24^

To our knowledge, this is the first study to examine the factors associated with complications of ALA in a predominant toddy‐drinking population of eastern India. There are a few limitations in our study, which include being a single‐center study and the inclusion of only indoor patients of gastroenterology ward in a tertiary care hospital.

In conclusion, the current study showed that older age, leukocytosis, hyperbilirubinemia, hypoalbuminemia, hyponatremia, chronic alcoholism, and smoking are independent factors significantly associated with complications in patients of ALA. Hypoalbuminemia, larger volume of abscess, and male gender are independent variables associated with the requirement of interventional therapy in the current study cohort.
